# Image quality optimization: dynamic contrast-enhanced MRI of the abdomen at 3T using a continuously acquired radial golden-angle compressed sensing acquisition

**DOI:** 10.1007/s00261-023-04035-4

**Published:** 2023-10-04

**Authors:** Jiangyang Pan, Xian Shao, Hui Liu, Yang Li, Qi Wang

**Affiliations:** 1 Department of Radiology, The Fourth Hospital of Hebei Medical University, Shijiazhuang, 050000 Hebei China; 2 Department of Anesthesiology, The Fourth Hospital of Shijiazhuang, Shijiazhuang, 050000 Hebei China

**Keywords:** Dynamic contrast-enhanced magnetic resonance imaging, Compressed sensing, Abdominal, Free breathing, Golden angle

## Abstract

**Introduction:**

The image quality of continuously acquired free-breathing Dynamic Contrast-Enhanced (DCE) golden-angle radial Magnetic Resonance Imaging (MRI) of abdomen suffers from motion artifacts and motion-related blurring. We propose a scheme by minimizing patients’ motion status from breathing as well as optimizing the acquiring parameters to improve image quality and diagnostic performance of DCE-MRI with Golden-Angle Radial Sparse Parallel (GRASP) sequence of abdomen.

**Methods:**

The optimization scheme follows two principles: (1) reduce the impact on images from unpredictable and irregulate motions during examination and (2) adjust the sequence parameters to increase the number of radial views in each partition. For the assessment of image quality, signal-to-noise ratio (SNR), contrast-to-noise ratio (CNR), the severity of radial artifact, the degree of image sharpness, and a visual scoring of image quality with a 5-point scale were assessed.

**Results:**

A total of 64 patients were included in this study before (16 men, 14 women, age: 54.9 ± 17.0) and after (18 men, 16 women, age: 58.6 ± 12.6) the optimization scheme was performed. The results showed that the SNR values of right and left lobe of liver in both plain phase and arterial phase were significantly increased (All *P* < 0.001) after the GRASP sequence been optimized. Significant improvements in CNR values were observed in the arterial phase (All *P* < 0.05). The significant differences in scores at each phase for visual scoring of image quality, noise of the right and left lobe of liver, radial artifact, and sharpness indicating that the image quality was significantly improved after the optimization (All *P* < 0.001).

**Conclusion:**

Our study demonstrated that the optimized scheme significantly improved the image quality of liver DCE-MRI with GRASP sequence both in plain and arterial phases. The optimized scheme of GRASP sequence could be a superior alternative to conventional approach for the assessment of liver.

## Introduction

Dynamic Contrast-Enhanced Magnetic Resonance Imaging (DCE-MRI) has been wildly used to non-invasively assess the microcirculatory perfusion and vascular permeability of the tumor, which has shown promising results in evaluating treatment response to chemoradiotherapy in liver cancer [1, 2]. In literatures’ report, the diagnostic performance of DCE-MRI-derived parameters may be influenced by the temporal and spatial resolution of images. So, a high image quality is very important in improving the performance of DCE imaging [3]. In the abdomen, the motion artifacts caused by respiratory movement and physiological peristalsis hinder the application of this technique in the abdomen. One approach to address breathing-related artifacts is using radial sampling of k-space [4]. A recently described acquisition technique combining a continuously acquired radial k-space trajectory with golden-angle radial sparse parallel (GRASP) reconstruction employing compressed sensing offers a new free-breathing imaging technique for dynamic imaging.

The GRASP sequence can acquire high spatial and high temporal resolution as well as motion robustness to DCE-MRI in liver imaging. Previous studies have shown that compared to the conventional breath-held Cartesian acquisition, GRASP can produce images with improved spatial resolution which can be used for diagnostic purposes [4]. However, researches also suggested some artifacts in abdominal imaging, especially when the DCE acquisition of golden-angle radial data is performed with free breathing, the reconstructed image shows high quality in the venous phase but slightly lower quality in the arterial phase [5]. This is probably caused by the transient dyspnea (a side effect of bolus injection in which the patient suffers from breathing disorder for around 20 s after bolus injection) that leads to respiratory motion artifacts in the arterial phase, which makes the reconstructed images less suitable for diagnosis [6]. The arterial phase in liver MRI is critical for the detection and characterization of focal liver lesions [7–10]. Therefore, the image quality of the arterial phase must be optimized without artifacts to allow accurate diagnosis of focal liver lesions. Besides, even though this method can reduce the streaking artifacts, it does not account for motion directly, which can subsequently lead to motion-related blurring. Since GRASP still suffers from residual respiratory blurring, it may lead to reduced vessel-tissue and lesion-tissue contrast, which influence the interpretation of the images and affect the quantification process in the case of a quantitative assessment of perfusion as provided by DCE-MRI.

There were a few studies evaluating the optimization scheme of DCE-MRI with GRASP sequence and have achieved good image quality in lung and pancreas [11, 12]. To overcome the above limitations of GRASP, we propose a scheme by minimizing patients’ motion status from breathing during examination as well as optimizing the acquiring parameters to improve image quality and diagnostic performance of DCE-MRI with GRASP sequence of abdomen. In our hypothesis, this optimization scheme could significantly improve the image quality in both plain and arterial phases, which may have important diagnostic value in the study of abdominal disease using DCE-MRI with GRASP sequence in future.

## Methods

### Participants

This retrospective study was performed at the radiological department of the Fourth Hospital of Hebei Medical University between Oct 2020 and Oct 2022. The methods and patients enrolled in the optimization of the diagnostic performance of DCE-MRI with GRASP sequence have referred to previously published articles [4, 13]. Inclusion criteria: (1) Outpatients who did not have history of radiotherapy and chemotherapy; (2) received DCE-MRI scans of the upper abdomen with free breathing; (3) received extracellular contrast agent; and (4) fasted for four hours before the examination. Exclusion criteria: (1) Patients with chronic liver diseases (cirrhosis, fatty liver, alcoholic liver, etc.) and (2) Patients with inconsistent sequence parameters. A total of 64 consecutive patients were included in this study before (16 men, 14 women, age: 54.9 ± 17.0) and after (18 men, 16 women, age: 58.6 ± 12.6) the optimization of the acquiring parameters. This study was approved by the institutional review board, with no requirement for individual informed consent.

### Acquisition and reconstruction

The GRASP technique used in this study combined a continuously acquired radial k-space trajectory with golden-angle sampling (~111.25°) and sparse parallel reconstruction employing compressed sensing, which offers simultaneous high spatial and high temporal resolution as well as motion robustness to DCE-MRI [14, 15]. DCE-MRI of abdomen with the GRASP sequence was performed at a 3T MR scanner (MAGNETOM Vida, Siemens Healthcare, Erlangen, Germany) with all patients in supine position. The acquired images were divided into two groups (before vs after optimization). The optimization scheme follows two principles: (1) reduce the impact on images from unpredictable and irregulate motions during examination and (2) adjust the sequence parameters to increase the number of radial views in each partition. Patients in optimization group were informed to remain calm and try to breathe easily before examination. To reduce the streak artifacts, arms were moved overhead with support. If applicable, a modest weight of sandbag would be placed upon patients’ abdomen to reduce the motion status in breathing. In order to increase the number of radial views, the scanning parameters of GRASP sequence were adjusted based on the following rules: (1) extend the total scanning time; (2) increase the slice thickness; (3) reduce the number of slices; (4) reduce the oversampling; and (5) shorten the time of repetition (TR). Details of the sequence parameters before and after optimization are presented in Table 1. In our institution, pre-contrast MRI was obtained for 23 seconds followed by GRASP scan during free breathing. For dynamic MRI, the conventional exam protocol consisted of a transversal T1-weighted (T1w) dixon breath-holding (BH) sequence (slice thickness = 3.5 mm, 15 s), a transversal fat-saturated (FS) T2w turbo spin echo (TSE) sequences (slice thickness = 6 mm, 2 min 20 s), a transversal diffusion-weighted imaging (DWI) (slice thickness = 6 mm, 2 min 32 s), GRASP sequence (6 min 1 s), and a post-contrast transversal T1w FS-BH sequence (slice thickness = 3.5 mm, 15 s). We optimized the sequence to transversal T1w dixon BH sequence, GRASP sequence, transversal T2w FS-TSE sequence, transversal DWI sequence, and post-contrast transversal T1w FS-BH sequence. In the optimized scheme, the DCE-MRI using the GRASP sequence was obtained continuously during free breathing for 5 minutes and 58 seconds (over 2500 radial spokes). The scan was performed during simultaneous injection of a standard dose of gadoteric acid (0.1 mmol/kg) administered at a rate of 2.5 mL/s.

**Table 1:** Scanning parameters of GRASP sequence before and after optimization

	Slice thickness	Slice numbers	FOV	Matrix	TR	TE	Radial views	Scanning time
Before	2.5	80	430	256	3.5	1.23	1423	6:01
After	4	48	380	256	2.8	1.26	2721	5:58

### MR image quantitative DCE analysis

Two radiologists (XX and XX) manually selected the best structured images at the unenhanced phase, early arterial phase, and late arterial phase among the reconstructed series for both groups of patients. The abdominal aorta was identified after contrast arrival when it had the highest signal intensity, whereas the portal vein was most clearly visible during the portal venous phase. Respectively, for each phase, three slices which best identified the structures of the main portal vein, left branch, and right branch of the hepatic portal vein were selected. Hence, 9 datasets from each patient were reviewed by readers. For the assessment of image quality, signal-to-noise ratio (SNR) of the left lobe and right lobe of the liver, defined as the ratio of the liver signal intensity (SI_liver_) and the background standard deviation (SD), were calculated as SNR = SI/SD. The contrast-to-noise ratio (CNR) of left lobe and right lobe of the liver, defined as the ratio of the absolute difference between SI_liver_ and spleen signal intensity (SI_spleen_) to the background standard deviation, were calculated as CNR= (SI_liverr_−SI_spleen_)/SD [16]. Without awareness of the scanning parameters, one radiologist (8-years experiences in interpreting gynecological cancers) assessed MR images regarding the severity of radial artifact, the degree of image sharpness and a visual scoring of image quality with a 5-point scale. The quality index was defined as follows: 1 (Extremely poor), 2 (Poor), 3 (Fair), 4 (Good), and 5 (Excellent), where indices 1 and 2 are considered clinically unacceptable and 3 to 5 clinically acceptable [17].

### Statistical analyses

Statistical analyses were performed using SPSS (Version 22; IBM, United States). All measurement values were tested for normal distribution using the Kolmogorov–Smirnov test. Continuous variables were presented as mean ± SD or median (range) depending on the normality of the data. Image quality parameters were compared using the independent *t* test or Mann–Whitney *U* test between the two groups. A 2-sided *P* value below 0.05 was considered as statistically significant.

## Results

**Fig. 1:**
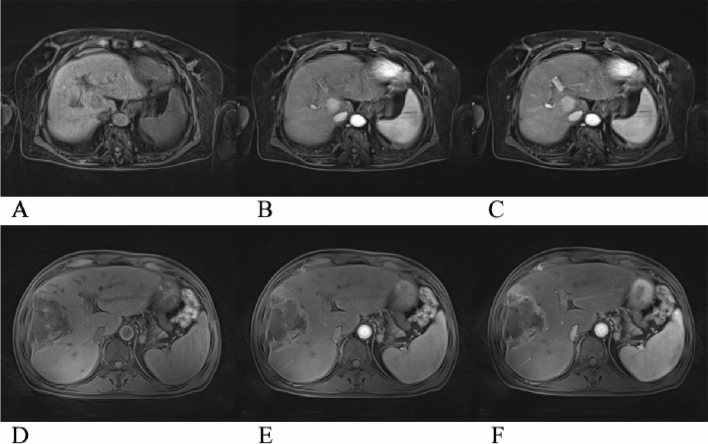
**A**–**C** Images before optimization, female, 64 years old with liver cancer and splenomegaly. In plain scan phase (**A**), the subjective image quality scores about noise of the left and right lobes of the liver, the degree of radial artifact, image sharpness, and the overall image quality were, respectively, 2, 2, 2, 3, and 2; in early arterial phase **B**: 3, 3, 2, 3, and 3; in late arterial phase **C**: 3, 3, 3, and 3. (**D**–**F**) Images after optimization, patient: male, 58 years old after interventional therapy for liver cancer and splenomegaly. In plain scan phase (**D**), the subjective image quality scores about noise of the left and right lobes of the liver, the degree of radial artifact, image sharpness, and the overall image quality were, respectively, 4, 5, 4, 4, and 5; early arterial phase **E**: 5, 5, 5, 4, and 5; late arterial **F**: 5, 5, 5, 4, and 5

Figure 1 shows the image comparisons of two patients who underwent abdominal DCE-MRI acquired with free-breathing acquisition and reconstruction with GRASP sequence before and after the optimization. The images of a 64-year-old female patient with liver cancer and splenomegaly (Fig. 1A–C) presented the relatively low subjective image quality scores (2–3 points) in plain scan phase, the early arterial phase, and the late arterial phase before optimization. And after optimization, the images of a 58-year-old male patient with liver cancer and splenomegaly (Fig. 1D–F) showed the satisfied subjective image quality scores (4–5 points) in plain scan phase, the early arterial phase, and the late arterial phase. As shown in the following sections, this optimization scheme led to similarly good results in other patients.

Table 2 presents the comparison of SNR before and after the optimization of DCE-MRI with GRASP sequence. The results showed that the SNR values of right and left lobe of liver in both plain phase and arterial phase were significantly increased (All *P* < 0.001) after the GRASP sequence been optimized. Additionally, this improvement was also observed in each individual slice. On average, SNR values were higher in late arterial phase compared to the plain and early arterial phase, and the SNR values in the right and left lobe of liver were similar in each phase and slice.

**Table 2:** Signal-to-noise ratio (SNR) of left lobe and right lobe of the liver before and after optimization in dynamic contrast-enhanced (DCE) magnetic resonance imaging (MRI) of liver with GRASP sequence

	SNRLL		P	SNRLR#		P
	After	Before		After	Before	
Plain						
Slice 1	156.21 (133.38,232.16)	79.13 (50.76,116.20)	<0.001	172.97 (149.39,213.55)	87.875 (58.67,149.98)	< 0.001
Slice 2	171.05 (132.85,222.67)	78.63 (46.87,120.35)	<0.001	179.03 (134.15,253.45)	88.16 (64.29,154.43)	< 0.001
Slice 3	145.91 (132.61,204.76)	81.26 (50.51,112.27)	<0.001	168.22 (129.71,245.04)	97.125 (61.86,127.21)	< 0.001
Early Arterial						
Slice 1	190.74 (154.34,378.53)	90.17 (80.13,156.99)	< 0.001	195.66 (171.91,326.32)	111.98 (92.74,163.29)	< 0.001
Slice 2	194 (147.07,257.16)	94.04 (58.01,154.89)	< 0.001	193.23 (153.81,336.16)	113.42 (73.3,187.84)	< 0.001
Slice 3	172.17 (152.1,245.3)	86.76 (58.41,135.52)	< 0.001	179.26 (142.87,293.33)	100.35 (64.98,178.29)	< 0.001
Late Arterial						
Slice 1	221.95 (174.76,310.05)	104.13 (87.51,173.73)	< 0.001	223.77 (196.1,302.18)	123.52 (101.79,198.62)	< 0.001
Slice 2	235.25 (184,444.65)	106.53 (72.5,193.3)	< 0.001	231.5 (187.43,358)	125.75 (93.18,209.68)	< 0.001
Slice 3	221.17 (173.95,403.11)	87.86 (63.5,153.37)	< 0.001	209.67 (163.51,328.53)	101.88 (75.04,183.88)	< 0.001

*SNRLL* signal-to-noise ratio of left lobe of the liver, *SNRLR* signal-to-noise ratio of right lobe of the liver

The comparison of the CNR values before and after the optimization of DCE-MRI with GRASP sequence is showed in Table 3. During the plain phase, the CNR values in both right and left lobe of liver were not differed significantly after the optimization of DCE-MRI with GRASP sequence (All *P* > 0.05). But significant improvements in CNR values were observed in the arterial phase (All *P* < 0.05). On average, CNR absolute values were higher in the late arterial phase compared to the plain and early arterial phase. No obvious difference on the average CNR values was observed between different slices and the sides of lobe.

**Table 3:** Contrast-to-noise ratio (CNR) of left lobe and right lobe of the liver before and after optimization in dynamic contrast-enhanced (DCE) magnetic resonance imaging (MRI) of liver with GRASP sequence

	CNRLL		P	CNRLR		P
	After	Before		After	Before	
Plain						
Slice 1	23.81 (− 13.07,55.41)	23.55 (9.44,36.23)	0.693	29.02 (2.2,52.5)	30.72 (22.25,57.62)	0.247
Slice 2	19.11 (− 1.94,54.06)	14.2 (5.15,36.61)	0.742	22 (4.13,76)	33.94 (23.4,55.58)	0.141
Slice 3	17.43 (− 0.11,59.91)	20.53 (4.97,40.68)	0.486	17.95 (2.91,47.52)	26.87 (15.86,51.89)	0.171
Early Arterial						
Slice 1	− 68.22 (− 129, -21.93)	− 18.9 ± 58.92	0.004	− 69.22 (-146.46,12.09)	1.83 (− 42.89,16.36)	0.008
Slice 2	− 81.43 (− 178.11,-37.14)	− 28.22 (− 52.32,2.84)	0.002	− 81.85 (-146.55,-19.31)	− 5.34 (− 35.3,10.96)	0.002
Slice 3	− 62.55 (− 160.63,-19.45)	− 21.69 ± 54.11	0.004	− 86.63 ± 106.17	− 12.12 ± 49.33	0.001
Late Arterial						
Slice 1	− 176.57 (− 373.64,-77.19)	− 72.17 (− 108.99,− 28.01)	0.002	− 194.7 (− 366.17,-86.35)	− 54.42 (− 80.49,− 26.25)	< 0.001
Slice 2	− 215.35 (− 358.18,-109.91)	− 79.08 (− 103.02,− 42.195)	< 0.001	− 186.19 (− 338.07,-117.5)	− 49.01 (− 80.63,− 31.91)	< 0.001
Slice 3	− 185.37 (− 318.89,-84.74)	− 61.82 (− 100,-33.12)	< 0.001	− 222.63 (− 286.4,-80.53)	− 43.76 (− 61.4,− 28.16)	< 0.001

*CNRLL* contrast-to-noise ratio of left lobe of the liver, *CNRLR* contrast-to-noise ratio of right lobe of the liver

A summary of qualitative reading scores is provided in Table 4. The results of visual scoring of image quality with a 5-point scale demonstrated poor to good, with significant difference before and after the optimization. The whole image quality scored as 3, 4, and 4 in plain phase, early arterial phase, and late arterial phase after the optimization, respectively. And the scores for whole image quality before optimization were 2, 2, and 3 in each phase, respectively. The significant differences in scores at each phase for noise of the right and left lobe of liver, radial artifact, and sharpness indicating that the image quality was significantly improved after the optimization (All *P* < 0.001). All subjective image quality scores ranged from 3 to 5 points, suggesting a fair to excellent image quality in each phase after optimization.

**Table 4:** Subjective image quality score about image noise, radial artifact, sharpness, and whole image quality before and after optimization in dynamic contrast-enhanced (DCE) magnetic resonance imaging (MRI) of liver with GRASP sequence

	Plain		*P*	Early arterial		*P*	Late arterial		*P*
	Before	After		Before	After		Before	After	
NoiseLL	2 (2,2)	3 (3,4)	< 0.001	2 (2,3)	4 (4,5)	< 0.001	3 (2,3)	4 (4,5)	< 0.001
NoiseRL	2 (2,2)	4 (3,4)	< 0.001	3 (2,3)	4 (4,5)	< 0.001	3 (3,3)	4 (4,5)	< 0.001
Radial artifact	2 (2,2)	3 (3,4)	< 0.001	2 (2,3)	4 (4,4)	< 0.001	3 (3,3)	4 (4,4)	< 0.001
Sharpness	2 (2,2)	3 (3,4)	< 0.001	3 (2,3)	4 (4,4)	< 0.001	3 (3,3)	4 (4,4)	< 0.001
Whole quality	2 (2,2)	3 (3,4)	< 0.001	2 (2,3)	4 (4,5)	< 0.001	3 (3,3)	4 (4,5)	< 0.001

*NoiseLL* noise of left lobe of the liver, *NoiseRL* noise of right lobe of the liver

## Discussion

In the current study, we assessed image quality and artifacts in the liver at a 3T MR scanner applying DCE-MRI of abdomen with the GRASP sequence. The results demonstrated that the optimized scheme significantly improved the image quality of liver DCE-MRI with GRASP sequence both in plain and arterial phases. These results may reflect, in part, the optimization role of reducing the impact on images from unpredictable and irregulate motions during examination and adjusting the sequence parameters to increase the number of radial views in each partition in contrast to conventional DCE-MRI with GRASP sequence. Therefore, the optimized scheme of GRASP sequence could be a superior alternative to conventional approach for the assessment of liver.

The optimized scheme with GRASP sequence showed higher scores in all qualitative assessments, including SNR, CNR, overall image quality, radial artifacts, sharpness, and noise of the liver. In this regard, the findings of our study are clinically relevant for imaging assessment of the number of radial views and respiratory motion artifacts from involuntary movement due to free breathing or swallowing. We believed maximizing the radial views and minimizing respiratory motion artifacts would be beneficial for the accurate evaluation of not only the primary lesions but also recurrences in postoperative follow-up MR imaging. Previous study suggested short breath-hold MR technique has better hepatic arterial phase image quality with a lower incidence of breath-hold difficulty. In light of this, we removed patients’ arms overhead with support and place a modest weight of sandbag upon patients’ abdomen to reduce the motion status in breathing [18]. In terms of the number of radial views, we recommended a total number of 2500 or above radial views in our experiences.

Although the conventional 2.5-mm slice thickness showed reasonable spatial resolution and clear details of the veins and their motion in patients’ MR image, we increased the slice thickness to from 2.5 to 4 mm in our optimization scheme in order to improve the missing slice artifact and intra‐bin variability, which can lead to an increased SNR. But this adjustment would potentially decrease the temporal resolution and create partial volume artifacts. As for the dynamic phases, a previous study found that the gated 6-second GRASP showed better diagnostic performance in detecting focal liver lesions [19]. In our institution, we used the gated 6-seconds GRASP to visualize more subtle enhancement characteristics in the early dynamic phase and contribute to improved diagnostic performance. A shorter temporal resolution would be able to depict the hemodynamic changes of focal liver lesions in greater detail and overcome the issue of temporal blurring. However, it must be noted that a decreased number of radial views per image and an increased slice thickness can increase the streak artifacts and susceptibility to motion as well as lower SNR value [4, 20]. Thus, we reduced the FOV from 430 mm to 380 mm, the number of slices from 80 to 56, as well as the TR time in our optimized scheme to balance the diagnostic performance at our best. In our study, since the k-space center is read out with every radial view, we extended the scanning period from 3 mins and 17 seconds to 5 mins 23 seconds to increase the number of radial views per image. However, imaging time should be clinically acceptable. Decreasing the imaging time should be more considered in future studies with patients, for example, by leaving out the other imaged acquisition plane or decreasing the FOV or number of slices to consist of smaller area of abdomen.

This study should be considered with limitations. Firstly, our sample size and number of liver lesions were insufficient to assess for differences in lesion detectability. Although the GRASP sequence may have increased diagnostic accuracy given an improved image quality, future studies with a larger sample size and a gold standard for the presence of lesions are required to further compare the diagnostic accuracies of the optimization scheme for liver lesion detection and characterization. Secondly, we did not measure the kappa values and perform test for interobserver agreement. But given the relatively constant scores assigned for most of the measures, with most scores equaling just one or two specific values, chances for the agreement between the readers was quite high. In addition, despite attempts to blind the readers to the imaging sequence during reading sessions, readily identifiable differences in the images before and after the optimization made it impossible to completely blind the readers. Thirdly, patients were not evaluated under both schemes. However, if patients were designed to undergo both schemes, a second application of contrast medium would have been necessary. This increased dose of contrast material would not have been justifiable.

## Conclusion

Our study demonstrated that the optimization role of reducing the impact on images from unpredictable and irregulate motions during examination and adjusting the sequence parameters to increase the number of radial views in each partition can improve the image quality in contrast to conventional DCE-MRI with GRASP sequence. Our optimized scheme significantly improved the image quality of liver DCE-MRI with GRASP sequence both in plain and arterial phases. The optimized scheme of GRASP sequence could be a superior alternative to conventional approach for the assessment of liver.

## Abbreviations

DCE-MRI: Dynamic contrast-enhanced magnetic resonance imaging
GRASP: Golden-angle radial sparse parallel
BH: Breath-holding
TSE: Turbo spin echo
DWI: Diffusion-weighted imaging
SNR: Signal-to-noise ratio
CNR: Contrast-to-noise ratio
SD: Standard deviation

## References

[1] Verma S, Turkbey B, Muradyan N, Rajesh A, Cornud F, Haider MA (2012). Overview of dynamic contrast-enhanced MRI in prostate cancer diagnosis and management. AJR American journal of roentgenology..

[2] Chen BB, Shih TT (2014). DCE-MRI in hepatocellular carcinoma-clinical and therapeutic image biomarker. World journal of gastroenterology..

[3] Hao W, Zhao B, Wang G, Wang C, Liu H (2015). Influence of scan duration on the estimation of pharmacokinetic parameters for breast lesions: a study based on CAIPIRINHA-Dixon-TWIST-VIBE technique. European radiology..

[4] Chandarana H, Feng L, Block TK, Rosenkrantz AB, Lim RP, Babb JS (2013). Free-breathing contrast-enhanced multiphase MRI of the liver using a combination of compressed sensing, parallel imaging, and golden-angle radial sampling. Investigative radiology..

[5] Motosugi U, Bannas P, Bookwalter CA, Sano K, Reeder SB (2016). An Investigation of Transient Severe Motion Related to Gadoxetic Acid-enhanced MR Imaging. Radiology..

[6] Chandarana H, Feng L, Ream J, Wang A, Babb JS, Block KT (2015). Respiratory Motion-Resolved Compressed Sensing Reconstruction of Free-Breathing Radial Acquisition for Dynamic Liver Magnetic Resonance Imaging. Investigative radiology..

[7] Goodwin MD, Dobson JE, Sirlin CB, Lim BG, Stella DL (2011). Diagnostic challenges and pitfalls in MR imaging with hepatocytespecific contrast agents. Radiographics..

[8] Park YS, Lee CH, Kim IS, Kiefer B, Woo ST, Kim KA (2014). Usefulness of controlled aliasing in parallel imaging results in higher acceleration in gadoxetic acid-enhanced liver magnetic resonance imaging to clarify the hepatic arterial phase. Investigative radiology..

[9] Ringe KI, Husarik DB, Sirlin CB, Merkle EM (2010). Gadoxetate disodium-enhanced MRI of the liver: part 1, protocol optimization and lesion appearance in the noncirrhotic liver. AJR American journal of roentgenology..

[10] Willatt JM, Hussain HK, Adusumilli S, Marrero JA (2008). MR Imaging of hepatocellular carcinoma in the cirrhotic liver: challenges and controversies. Radiology..

[11] Oyama K, Ichinohe F, Yamada A, Kitoh Y, Adachi Y, Hayashihara H (2022). Optimal Temporal Resolution to Achieve Good Image Quality and Perform Pharmacokinetic Analysis in Free-breathing Dynamic Contrast-enhanced MR Imaging of the Pancreas. Magnetic resonance in medical sciences : MRMS : an official journal of Japan Society of Magnetic Resonance in Medicine..

[12] Chen L, Liu D, Zhang J, Xie B, Zhou X, Grimm R (2018). Free-breathing dynamic contrast-enhanced MRI for assessment of pulmonary lesions using golden-angle radial sparse parallel imaging. Journal of magnetic resonance imaging : JMRI..

[13] Kaltenbach B, Bucher AM, Wichmann JL, Nickel D, Polkowski C, Hammerstingl R (2017). Dynamic liver magnetic resonance imaging in free-breathing: feasibility of a Cartesian T1-weighted acquisition technique with compressed sensing and additional self-navigation signal for hard-gated and motion-resolved reconstruction. Investigative radiology..

[14] Winkelmann S, Schaeffter T, Koehler T, Eggers H, Doessel O (2007). An optimal radial profile order based on the Golden Ratio for time-resolved MRI. IEEE transactions on medical imaging..

[15] Feng L, Grimm R, Block KT, Chandarana H, Kim S, Xu J (2014). Golden-angle radial sparse parallel MRI: combination of compressed sensing, parallel imaging, and golden-angle radial sampling for fast and flexible dynamic volumetric MRI. Magnetic resonance in medicine..

[16] Welvaert M, Rosseel Y. On the definition of signal-to-noise ratio and contrast-to-noise ratio for FMRI data. PloS one. 2013;8(11):e77089. 10.1371/journal.pone.0077089 10.1371/journal.pone.0077089 PMC3819355 24223118

[17] Martinez JA, Moulin K, Yoo B, Shi Y, Kim HJ, Villablanca PJ (2020). Evaluation of a Workflow to Define Low Specific Absorption Rate MRI Protocols for Patients With Active Implantable Medical Devices. Journal of magnetic resonance imaging : JMRI..

[18] Yoo JL, Lee CH, Park YS, Kim JW, Lee J, Kim KA (2016). The Short Breath-Hold Technique, Controlled Aliasing in Parallel Imaging Results in Higher Acceleration, Can Be the First Step to Overcoming a Degraded Hepatic Arterial Phase in Liver Magnetic Resonance Imaging: A Prospective Randomized Control Study. Investigative radiology..

[19] Yoon JH, Lee JM, Yu MH, Hur BY, Grimm R, Block KT (2018). Evaluation of Transient Motion During Gadoxetic Acid-Enhanced Multiphasic Liver Magnetic Resonance Imaging Using Free-Breathing Golden-Angle Radial Sparse Parallel Magnetic Resonance Imaging. Investigative radiology..

[20] Chandarana H, Block TK, Rosenkrantz AB, Lim RP, Kim D, Mossa DJ (2011). Free-breathing radial 3D fat-suppressed T1-weighted gradient echo sequence: a viable alternative for contrast-enhanced liver imaging in patients unable to suspend respiration. Investigative radiology..

